# Instrumental Delivery*Read at Meeting of Central Texas Medical Association, at Waco, Texas, January 10, 1900

**Published:** 1900-03

**Authors:** A. R. Kuykendall

**Affiliations:** Morgan, Texas


For Texas Medical Journal.

### Instrumental Delivery.*

BY DR. A. R. KUYKENDALL, MORGAN, TEXAS.

According to the distinguished author and teacher, Prof. Hugh S. Hodge, there are four subdivisions of instrumental delivery, viz.: (1) In which the instruments are to be applied to the child, so as not to injure its tissues, and at same time to accomplish the process of parturition with safety to both mother and child. (2) Where the instruments are applied to child—upon the supposition that its death, if not actually occurred, is inevitable—with a view of diminishing its size to such an extent that its delivery may be accomplished. This operation is known as craniotomy, evisceration, etc. (3) Where operation is performed not on child, but on mother,

*Read at Meeting of Central Texas Medical Association, at Waco, Texas, January 10, 1900.

with the hope that both child and mother will be preserved, which procedure is known as symphisiotomy and Cæsarian section. (4) mature labor, which is chiefly for the purpose of saving the mother, with a reasonable hope for the child, if not performed too early in pregnancy.

You will see that the subject of instrumental delivery is quite extensive, and believing that an attempt to speak of it in all its parts would be too tiresome to you on this occasion, I desire to dismiss from further consideration all reference to the subject, except to the obstetric forceps, the introduction of which, to medical men, I regard as one of the greatest, indeed the greatest, boon that can be recounted by the physician and surgeon.

There are very few women in the agony of child birth who will not accept some means tending to shorten the duration of this suffering. And what physician who, having this means at his command, would not readily resort to instrumental delivery when the condition of mother or child was perilous?

I acknowledge that doubtless there have been many mothers and many children made to give up their lives by the attempt of the use of the obstetric forceps by incompetent operators. It is true, also, that there have been failures to deliver women with forceps, due to unrecognized complications, such as narrow and deformed pelvis, obstructions by tumors, abnormal development of child, etc. Yet these cases and accidents are too rare in this age of our progress to induce us to abandon their use, or to refuse to accept them as our greatest obstetric aids.

The weight of the child, the uterine contractions and the mother's efforts are nature's means of bringing forth the unborn—but poor nature often becomes helpless in her efforts, when it not only behooves, but fittingly becomes the attendant, to offer her his safe and best assistance, namely, the obstetric forcep.

In the absence of the proper appreciation of the merits of this instrument, we find physicians jeopardizing the life of both mother and child, depending wholly upon the physical strength of frail and delicate and exhausted woman to conduct a warfare against a foe whose forces are mighty, indeed too often greater than nature's powers of resistance. Again, there are men of our profession who are not patient enough to wait and trust to nature's good and faithful efforts, and who, discarding the use of the forceps, must do something to excite pains, resort to artificial uterine stimulants, such as ergot, quinine, strychnine, and the like, thereby increasing the poor woman's agony, increasing danger, even jeopardizing the life of both child and mother. *Don't give ergot* to hasten pains and shorten duration of labor, but use the forceps, which should always be looked upon as a helping remedy, rather than a dangerous resort. The obstetric forceps is not to be regarded only as a means to assist labor, or to hastily terminate the agonizing pains of the mother, but as an instrument beneficial alike to both child and mother. Often is forcep delivery demanded to save the life of the child. So, also, the mother in convulsions, excessive hemorrhage, rupture of uterus, arrest of the progress of labor, rigor perineum, etc.

I do not care to speak of the different kinds and makes of forceps, or advise the use of any particular make. I have never owned or used any other than the long handled Hodge forceps, which I have found distinctively ample in my hands for the purposes for which the obstetric forceps was intended. This instrument may be applied to the fore coming or after coming head, or to the pelvis in the breach presentations. In the latter condition, however, it is seldom necessary, and is condemned by some able writers. My method of the application of forceps to the vertex is as follows: Having diagnosed the position, I hold forceps in front of me in that position occupied by the fœtal head, which serves somewhat as a guide to the correct placing of the instrument. The mother should be anæsthetized to the degrees known as obstetric anæsthesia, that is, not to complete, or surgical anæsthesia. She should be placed in a convenient position for the operator. If there is suspicion of infection of the parturient parts, these should be cleansed with douche of sterilized salt solution or solution of bichloride. Instruments and hands should be thoroughly sterilized, and anointed with warm sterilized castor oil or vaseline. With fingers of the right hand as a guide, first introduce the left or male blade into the sacro-iliac fossa and then adjust the blade to side of fœtal head. The right or female blade is next introduced in like manner, the fingers of the left hand acting as directors. When both blades are thus introduced, "rotate that blade which corresponds with the diagonal diameter of the pelvis occupied by the fœtal head." Always apply forceps to the fœtal head and not in reference to sides of the pelvis. Having rotated the blade necessary to locking, we should make gentle compression until the fenestræ fit snugly to sides of head. When forceps are securely locked, place one or two fingers of left hand against vertex, and with the right hand make gentle but sufficient traction to insure against forceps slipping, thereby saving the rupture of the tissues of vertex, and a just censure to the operator. The degree of compression beyond this must be regulated by the necessities of the particular case, and by the judgment of the operator. Excessive and long continued compression endangers the life of the child, yet we know that when there is good, full placental pulsation, the fœtus can bear greater and longer continued compression. It should be borne in mind, too, that compression on the head by the forceps lessens that made by the head on the maternal soft parts, and thereby contributes to their integrity and safety.

Traction should always be made in the line of the axis of the pelvis. In the high forcep operation, the first traction is made backward and downward. In other words, in a line perpendicular to the autero-posterior diameter of the brim. When head reaches the pelvic floor, the traction of forceps should be upward and forward. It is not a safe procedure to resort to oscilatory manipulations, for fear of bruising and abrading the maternal soft parts. Yet doubtless there are cases wherein this maneuver seems necessary, and may be attempted without material damage.

Traction should be made gently and with sufficient weight to aid the head in its downward progress. In manipulating the forceps, grasp them loosely and allow the movements of the child to be imparted to the hand. Traction should be intermittent. If the head wants to recede, let it do so. If it wants to rotate, let it rotate, in fact help it to rotate. I regard the forceps as an instrument, the proper use of which will greatly assist the operator in preventing the tear of the perineum. When the presenting part, especially a large head, engages the perineum, the physician can control the weight and force of the pains against the perineum if he has his forceps applied. By thus controlling the pressure for a few moments, we give these delicate tissues time to so yield that the passage of the head does little or no damage.

In removing forceps, we should wait till the perineum is quite yielding, and in fact the head should be well engaged in the outlet when blades should be unlocked and removed hurriedly but gently.

In conclusion, it might be well enough to mention some of the conditions under which forceps can be used: "Pelvis must be normal or but slightly narrow. The antero-posterior diameter of the brim should not be less than three inches. The soft parts must be yielding. Os uteri must be fully dilated, or nearly so. Membrane must be ruptured. Head must or ought to be fixed and not floating above the brim of pelvis.

